# Chitosan/Calcium–Alginate Encapsulated Flaxseed Oil on Dairy Cattle Diet: In Vitro Fermentation and Fatty Acid Biohydrogenation

**DOI:** 10.3390/ani12111400

**Published:** 2022-05-29

**Authors:** Maghsoud Besharati, Ilias Giannenas, Valiollah Palangi, Tugay Ayasan, Fatemeh Noorian, Aristide Maggiolino, Jose Manuel Lorenzo

**Affiliations:** 1 Department of Animal Science, Ahar Faculty of Agriculture and Natural Resources, University of Tabriz, Ahar 5451785354, Iran; fatemeh.noorian08@gmail.com; 2 Laboratory of Nutrition, Faculty of Veterinary Medicine, Aristotle University of Thessaloniki, 54124 Thessaloniki, Greece; igiannenas@vet.auth.gr; 3 Department of Animal Science, Agricultural Faculty, Ataturk University, Erzurum 25240, Turkey; valiollah.palangi12@ogr.atauni.edu.tr; 4 Department of Organic Farming Business Management, Kadirli Faculty of Applied Sciences, University of Korkut Ata, Osmaniye 80000, Turkey; tayasan@gmail.com; 5 Department of Veterinary Medicine, University of Bari A. Moro, 70010 Valenzano, Italy; aristide.maggiolino@uniba.it; 6 Centro Tecnológico de la Carne de Galicia, Avd. Galicia 4, Parque Tecnológico de Galicia, 32900 Ourense, Spain; 7 Facultad de Ciencias de Ourense, Área de Tecnología de los Alimentos, Universidade de Vigo, 32004 Ourense, Spain

**Keywords:** biohydrogenation, calcium alginate, capsulation, chitosan, fatty acids, flaxseed oil

## Abstract

**Simple Summary:**

Most unsaturated fatty acids in the ruminant’s diet are hydrogenated in their rumen so that the composition of the fatty acids entering the rumen and their output is significantly different. Therefore, minimizing the ruminal biohydrogenation process of unsaturated fatty acids is one of the most important issues for feed supplements manufacturers and animal nutritionists to increase the availability of these fatty acids in the intestine. In recent years, encapsulation has been used to preserve the active ingredient in livestock; it is a method used to control the release of feed additives during digestion. There is a clear need to find a more effective method by which unsaturated fatty acids present in fat supplements can be protected to bypass rumen environment and its biohydrogenation, without negative effect on digestive efficiency, and be available in lower digestive tracts. For these reasons, this study aims to evaluate the use of natural materials to encapsulate fats and their effect on in vitro fermentation and fatty acid biohydrogenation. The results indicated that the percentage of ruminal saturated fatty acids decreased by encapsulation of flaxseed oil with chitosan (14% and 7%). The percentage of oleic unsaturated fatty acid by encapsulating flaxseed oil with chitosan (14%) had a significant increase compared to the control treatment (*p* < 0.05). Encapsulation of flaxseed oil with chitosan (14%) reduced the unsaturated fatty acids of ruminal biohydrogenation.

**Abstract:**

The aim of this study was to investigate the effect of using chitosan nanoparticles and calcium alginate in the encapsulation of flaxseed oil on the biohydrogenation of unsaturated fatty acids and in vitro fermentation. The experiments were performed in a completely randomized design with 7 treatments. The experimental treatments included: diets without oil additive (control), diet containing 7% flaxseed oil, diet containing 14% flaxseed oil, diet containing 7% oil encapsulated with 500 ppm chitosan nanocapsules, diet containing 14% flaxseed oil encapsulated with 1000 ppm chitosan nanocapsules, diet containing 7% of flaxseed oil encapsulated with 500 ppm of calcium alginate nanocapsules, diet containing 14% flaxseed oil encapsulated with 1000 ppm calcium alginate nanocapsules. The results showed that encapsulation of flaxseed oil with calcium alginate (14%) had a significant effect on gas production (*p* < 0.05). The treatment containing calcium alginate (14%) increased the digestibility of dry matter compared to the control treatment, but the treatments containing chitosan caused a significant reduction (*p* < 0.05). The results indicated that the percentage of ruminal saturated fatty acids decreased by encapsulation of flaxseed oil with chitosan (14% and 7%). The percentage of oleic unsaturated fatty acid by encapsulating flaxseed oil with chitosan (14%) had a significant increase compared to the control treatment (*p* < 0.05). As a result, encapsulating flaxseed oil with chitosan (14%) reduced the unsaturated fatty acids generated during ruminal biohydrogenation.

## 1. Introduction

The importance of using fats as an energy source in the diets of high-yielding ruminants, especially dairy cows at the beginning of lactation, has long been known for its negative energy balance [1,2,3]. In addition, the physiological importance of some unsaturated fatty acids has led to the targeted use of various fatty acids regardless of energy supply [4]. Nowadays, the use of oils and oilseeds in animal feed has been considered by researchers due to numerous biological roles. Reducing energy demand in livestock by reducing milk fat at the beginning of lactation by certain fatty acids [5,6,7], increasing the nutrition of carbohydrates and fats to meet the metabolic needs of livestock [8,9], and the tendency to increase the concentration of conjugated linoleic acid in livestock products due to its effects on human health [10,11,12] can be cited as some of the reasons for this approach. However, when unprotected oils are fed to cows, there is an extensive hydrolyzation and biohydrogenation by ruminal microorganisms that results in marginal increases in passage to intestine [13]. Moreover, potential detrimental side effects must be considered, such as altered rumen biohydrogenation pathways associated with alteration in digestive efficiency [14].

Biohydrogenation is the process in the rumen during which hydrogen is added to the double bonds of unsaturated fatty acids [15,16]. Biohydrogenation is mainly performed by rumen bacteria, while it is not well known the protozoa role [17,18]. However, it is accepted that ciliate protozoa are not directly involved in biohydrogenations activity [19], but actively contribute to lipolysis and influence biohydrogenation by different mechanisms. It seems that ciliate protozoa ingest and directly incorporate dietary PUFA in their cellular membranes, protecting them from hydrogenation by bacteria [20]. Moreover, recent studies reported that rumen protozoa have a high content of vaccenic acid and conjugated linoleic acid [21], suggesting that they may give a significant contribution to these acids flow from the rumen [22]. Most unsaturated fatty acids in the ruminant’s diet are hydrogenated in their rumen so that the composition of the fatty acids entering the rumen and their output is significantly different.

Therefore, minimizing the ruminal biohydrogenation process of unsaturated fatty acids is one of the most important issues for feed supplements manufacturers and animal nutritionists to increase the availability of these fatty acids in the intestine [23,24]. There are several ways to protect fats and oils from rumen biohydrogenation. Many techniques have been studied and developed to bypass ruminal biohydrogenation and degradation of unsaturated fatty acids. One of them is the utilization of rumen inert as their supplementation as calcium salts (although it provides little to no protection to polyunsaturated fatty acids). Differently, rumen-protected oils may represent an opportunity to achieve this goal [25]. The use of new techniques, such as microencapsulation of fatty acids in the last decade, has been used by food industry researchers as an effective method to protect unsaturated fatty acids from oxidation and reduce unpleasant odors and tastes [26].

Encapsulation is when a substance or several substances are coated with or trapped within a substance or system. In recent years, a technology called encapsulation has been used to preserve the active ingredient in livestock and to create a proper product quality and effectiveness; it is a method used to control the release of feed additives during digestion. In recent years, studies have been done on encapsulation and its effect [27,28,29].

In studies on encapsulated plant application in livestock production, it has been emphasized that encapsulation application can be applied as a good strategy to increase the potency of herbal components that can be used instead of synthetic antioxidants and AGF [30,31]. Like the antibacterial activity in the studies, it was determined that the antioxidant activity increased as a result of chitosan encapsulation [32].

Trends in demands for safer products have encouraged a search for natural alternatives to direct fed antibiotics. Chitosan (an N-acetyl-d-glucosamine polymer) is a natural non-toxic, biodegradable biopolymer [33] derived from deacetylation of chitin, a major component of the shells of crustaceans. The antimicrobial activity of chitosan has been noted as one of its most interesting properties [34,35], which has led to evaluation of its use in ruminant nutrition [36]. Apart from its biodegradable character in physiological conditions, chitosan has reactive amine and hydroxyl groups, which offer possibilities of graft reactions (i.e., carboxymethyl chitosan) and ionic interactions [37]. Chitosan is polycationic at pH less than 6 and reacts easily with negatively charged compounds such as proteins, anionic polysaccharides, fatty acids, and phospholipids, which can structure and texture products that Chitosan is used in their production to affect. Due to the presence of amino groups in the structure of chitosan, this substance has a better solubility in acidic environments.

Alginic acid, or sodium and potassium alginates (ALG), is one of the biomaterials of the adhesive mucosa due to its cell compatibility, biocompatibility, biodegradability, SOL-GEL transfer properties, and chemical versatility, which allows further modifications. To make it possible to adapt its properties, it has been studied for drug delivery. This substance is prepared from different types of seaweed. The many benefits of this material have led to an increase in the interest of the scientific community in alginate as a platform for the development of new nanodrug delivery systems in recent decades [38]. One of the most useful properties of alginate is its ability to crosslink in aqueous solutions by a mechanism through the carboxylic acid moiety of G units with calcium ions and in divalent cations (such as Ba^2+^, Ca^2+^, Zn^2+^) to form a three-dimensional network. It has been used for over 3 decades to encapsulate a wide range of drugs, proteins, genes, and cells.

In the light of all this information, there is a clear need to find a more effective method by which unsaturated fatty acids present in fat supplements can be protected to bypass rumen environment and its biohydrogenation, without negative effect on digestive efficiency, and be available in lower digestive tracts. For these reasons, this study aims to evaluate the use of natural materials to encapsulate fats and their effect on in vitro fermentation and fatty acid biohydrogenation.

## 2. Materials and Methods

Chitosan medium molecular weight (75–85% degree of distillation, CAS # 9012-76-4), tripolyphosphate (CAS # 7758-29-4, industrial grade) and sodium alginate were purchased from Sigma-Aldrich (Sigma-Aldrich Chemie GmbH, Eschenstr. 5, 82024 Taufkirchen, Germany).

Experimental treatments were (1) control (diet without oil), (2) diet with 7% flaxseed oil, (3) diet with 14% flaxseed oil, (4) diet with 7% encapsulated flaxseed oil (500 ppm chitosan nanocapsule, (5) diet with 14% encapsulated flaxseed oil (1000 ppm chitosan nanocapsule, (6) diet with 7% encapsulated flaxseed oil (500 ppm calcium alginate nanocapsule), and (7) diet with 14% encapsulated flaxseed oil (1000 ppm calcium alginate nanocapsule.

The diet contains alfalfa hay (21%), maize silage (19%), beet pulp (7%), wheat bran (2%), and dairy cattle concentrate (51%). The chemical composition of diet was DM 59%, CP 20%, NDF 38%, and ADF 20% (Table 1). Dairy cattle concentrate was purchased from feed manufactory (Eris^®^ Animal Feed Manufacture, Ahar, Iran).

### 2.1. Preparation of Oil-Loaded Chitosan and Calcium Alginate Nanoparticles (NPs)

The chitosan and calcium alginate nanoparticles were prepared according to methods described by Hosseini et al. [39] and Keawchaoon and Yoksan [40], with some modifications. Chitosan NPs and calcium alginate NPs were prepared separately using the oil-in-water emulsion technique in chitosan and calcium alginate solutions. Droplet solidification was carried out by pentasodium tripolyphosphate (PTP) solution to achieve nanoparticles (NPs) through the ionic gelation method. Briefly, two concentrations of chitosan and calcium alginate (1000 and 2000 ppm) in glacial acetic acid (1% (*v*/*v*)) were produced by stirring at room temperature (25 °C) for 12 h to the formation of aqueous phases. Büchner funnel and Whatman 42 paper were used to filtrate solutions after pH adjustment to 4.6 using NaOH (0.1 N). Then, Tween 80 (1%, *w*/*v*) was added to the aqueous solutions as a surfactant and stirred at 25 °C for 30 min to achieve homogeneous mixtures. The different levels of flaxseed oil (7 and 14%) were then gradually dropped in solutions prepared by chitosan and calcium alginate to produce four different mass ratios of chitosan to oil (500:7 and 1000:14) and calcium alginate to oil (500:7 and 1000:14). At the same time, the agitation (700 rpm for 10 min) was carried out at room temperature to produce oil-in-water emulsions. PTP (0.3%, *w*/*v*) was then prepared in distilled water and flush-mixed with prepared emulsions to obtain two mass ratios of chitosan to PTP and calcium alginate to PTP of 1:1. Subsequently, the mixtures were held to agitation at 25 °C for 30 min to effect crosslinking. The same method without flaxseed oil addition was used for unloaded nanoparticles. The produced particles were collected by centrifuge (SIGMA 8K, Germany) at 10,000× *g* for 35 min (4 °C) and washed five times with Tween 80 solution 1% (*v*/*v*), then dispersed in distilled water and treated by ultrasonic homogenizer (TOPSONICS, UP400, Iran) at 60 W (6 min) with a sequence of 3 s sonication and 7 s rest. The obtained dispersions were then freeze-dried and stored until further analysis (for measuring the particle size and zeta potential).

### 2.2. Measurement of Particle Size and Zeta Potential

A dynamic light scattering (DLS) instrument measured the mean particle size and zeta potential of freshly prepared chitosan, and calcium alginate NPs were measured by a dynamic light scattering (DLS) instrument (Zetasizer Nano ZS90, Malvern, UK). Results were represented as the means of three measurements ± standard deviation. The Zeta potential of chitosan and calcium alginate nanoparticles was evaluated at pH 4.6 and 25 °C. One mg of NPs were added in 10 mL of distilled water to the preparation of nanoparticles stock solution. According to Dilbaghi et al. [41], clear zeta cells (disposable) with 15 runs and equipoise time of 2 min were used for scanning of 1 mL stock solution.

### 2.3. Encapsulation Efficiency

The percentage of encapsulated flaxseed oil for chitosan NPs and calcium alginate NPs were determined by ultraviolet-visible (UV-vis) spectrophotometry (Shimadzu UV 2450, Japan) according to Chopra et al.’s [42] method after centrifugation the mixture at 11,000 rpm and unbounded flaxseed oil in the supernatant were estimated by utilizing UV-Visible spectrophotometer at 205 nm.

(1) $$\%\;\mathrm{Encapsulation}\;\mathrm{Efficacy}=\frac{\mathrm{Total}\;\mathrm{flaxseed}\;\mathrm{oil}-\mathrm{Unbounded}\;\mathrm{flaxseed}\;\mathrm{oil}}{\mathrm{Total}\;\mathrm{flaxseed}\;\mathrm{oil}}\times 100$$

### 2.4. Fourier Transform Infrared (FTIR) Characterization

The structural properties of chitosan and calcium alginate nanoparticles were analyzed at 25 °C by utilizing FTAR spectra (Bruker Co., Germany). The samples were mixed at the ratio of 1:100 with potassium bromide and pressed into a pallet for analysis. The spectra had a 4 cm^−1^ resolution and was recorded from 400 to 4000 cm^−1^.

### 2.5. In Vitro Gas Production Technique

The method of Fedorah and Hrudey [43] was used to measure gas production. First, the feedstuffs (all experimental treatments) were ground by a mill with a sieve diameter of 1 mm. The amount of 300 mg of each ground food was carefully weighed and transferred to 50 mL sterile serum bottles, and 5 repetitions were considered for each treatment for a total of 35 runs. Rumen fluid was prepared from 3 slaughtered sheep, mixed in a unique rumen fluid, and after straining by a four-layer mesh cloth inside the thermos flask (previously filled with sterile distilled water at 39 °C to avoid thermal shock to rumen fluid) and insufflating CO_2_ to ensure the anaerobic environment, it was immediately transferred to the laboratory. After transport, it was mixed and blended under a CO_2_ headspace for 30 s to remove any additional particles and/or attached organisms and then strained through 6 layers of cheesecloth [44]. Before transferring ruminal fluid into serum bottles, it was mixed with a buffer prepared by McDougall [45] in a ratio of 1:2 (ruminal fluid: buffer). In each bottle containing 300 mg of each experimental treatment, 20 mL of the mixture of ruminal fluid and buffer was added, and after anesthesia inside the bottle by infusion carbon dioxide gas, the bottle lid was closed with a rubber cap and metal press. It was tightly closed. All glasses were transferred to the shaker incubator at 120 rpm and at a temperature of 39 °C to measure the produced gas, and the operation of reading and recording the amount of gas produced due to food fermentation by Fedorah and Hrudey [43] method (gas volume) in 2, 4, 6, 8, 12, 24, 36, 48, 72, and 96 h after incubation. Ingredients and chemical composition of the experimental diet are shown in Table 1.

### 2.6. In Vitro Digestibility

This experiment was performed based on the gas production method. In this method, 15 replicates were prepared for each of the available treatments, and at 2, 4, 8, 12, and 24 h. Three replicates of each treatment were removed from the incubator, and all their contents were poured into laboratory falcons. All analysis for nutrient parameters performed before and after digestion and percentage of disappearance for each parameter was calculated as: (PB-PA)/PB, where PB is the quantity (g/kg) of parameter in the samples before the digestion and PA is the quantity (g/kg) of the parameter after digestion. Results were expressed as percentage [46].

Dry matter (DM) was determined using standard procedures [47] (method 930.15). Ash was determined by standard procedures [47] (method 942.05) using a muffle furnace at 550 °C for 16 h. Fat was determined using the Soxhlet extraction procedure [47] (Method 991.36), crude protein (CP) was determined by Kjeldahl N × 6.25 procedures [47] (Method 968.06). Neutral detergent fiber (NDF) and acid detergent fiber (ADF) were determined with the ANKOM fiber analyzer according to Van Soest et al. [48] and was corrected for residual acid-insoluble ash.

### 2.7. In Vitro Biohydrogenation

This experiment was also performed according to the method for measuring gas production and laboratory digestibility, with the difference that 9 repetitions are considered for each treatment and three replicates of each treatment are removed from the incubator at 2, 4, and 24 h. Their contents are stored at −20 °C to determine ruminal biohydrogenation [49], which is described below.

#### 2.7.1. Preparation of Samples for Determination of Fatty Acid Profiles

##### Fatty Acid Extraction

Azadmard-Damirchi and Dutta;s [50] method was used to extract fatty acid from experimental treatments left over from incubation. In short, the procedure was as follows.

The ruminal fluid and the remaining sample after incubation were poured into an Erlenmeyer flask and 25 mL of 1: 1 chloroform solution was added to methanol. A magnet was then placed in each Erlenmeyer to accelerate homogenization and extraction and mixed for 10 min. Then, 60–65 mL of the above solution was added into each Erlenmeyer flask and stirred for 1 h every 5 min.

Erlenmeyer was kept at room temperature, then Erlenmeyer contents were filtered using a Buchner funnel and filter paper. The remaining water was separated using a Buchner funnel and the oil and solvent layer was separated using an evaporator. The extracted oils were stored at −20 °C for later use [50].

##### Methylation Procedure

About 10 mg of fat was dissolved in 0.5 mL of n-hexane in the test tube and then 2 mL of 0.01 M NaOH was added to the dry methanol. The test tubes containing these solutions were kept in a 60 °C water bath for 10 min. Next, it was kept in room air for 10 min, and after the reaction, the test tube was placed under cold water, and 2 mL of 20% salt solution and 1 mL of n-hexane were added. After complete mixing, it was centrifuged at 2000 rpm for 5 min and the hexane layer containing the fatty acid methyl ester derivative was separated [50]. The fatty acid profile was determined with the model GC-mas (Agilent Technologies 7890B).

For GC–MS analysis, an Agilent 6890 gas chromatography with a 30 m to 0.25 mm HP-5MS capillary column coupled with an Agilent 5973 mass spectrometer (Agilent Technologies, Palo Alto, CA, USA) operating in EI mode at 70 eV was used. The injector and detector ports temperatures were set at 250 and 150 °C, respectively. Initially, the column temperature was held at 60 °C for 3 min and then was increased at a rate of 5 °C /min to 220 °C. The temperature of the column was held at 220 °C for 10 min.

### 2.8. Statistical Analysis

The obtained data were analyzed in a completely randomized statistical design according to the Proc mixed procedure of the SAS. To compare the means, Duncan’s multiple range was used. Significance was set at *p* < 0.05, and the results were expressed as means and mean standard error. All the analysis was performed using SAS 9.1 software (2018).

## 3. Results

### 3.1. Capsulation efficiency

In the present study, the micro coating efficiency was 87.47% for 500 ppm chitosan, 67.45% for 1000 ppm chitosan, 74.5% for 500 ppm calcium alginate, and 53.28% for 1000 ppm calcium alginate (Table 2).

### 3.2. Zeta Potential

The zeta potential values for 500 and 1000 ppm of chitosan and 500 ppm and 1000 ppm of calcium alginate are shown in Table 2, for each of +56.2 mV, +45.5 mV, and +0.9 mL, respectively. Volts and +0.6 mV were measured.

### 3.3. Particle Size Distribution

**Figure 1:**
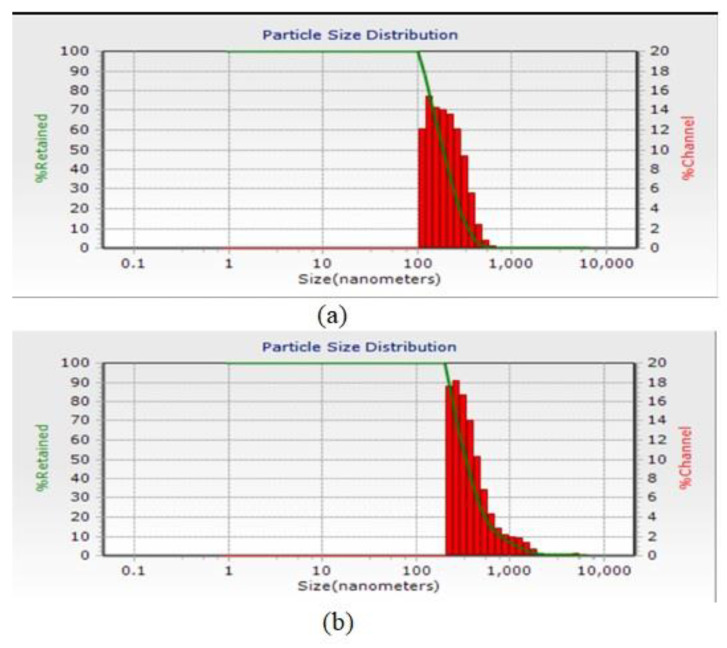
Particle size distribution diagram of microcapsules after formation: (**a**) chitosan nanoparticles and (**b**) calcium alginate nanoparticles.

The results of the DLS test for the average numerical size of nanoparticles formed using 500 ppm chitosan and 500 ppm calcium alginate are shown in Figure 1, which are 190.2 nm and 334 nm, respectively. Because encapsulation efficiency and zeta potential were better for 500 ppm treatments, particle size distribution tests and infrared spectroscopy were considered only for these treatments.

### 3.4. Fourier Transform Infrared Spectroscopy Test (FTIR)

**Figure 2:**
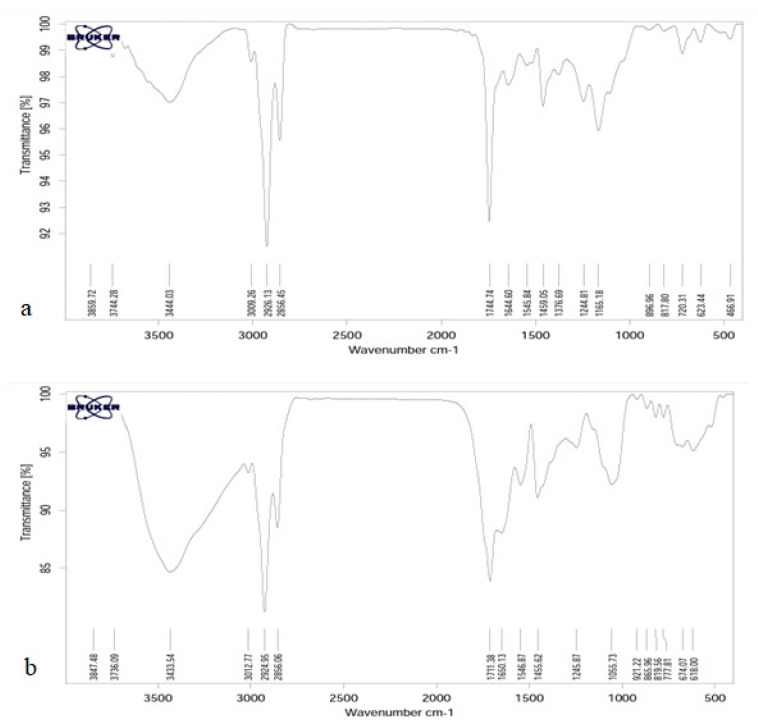
FTIR spectra of calcium alginate (**a**) and chitosan (**b**).

The results of FTIR spectroscopy, chitosan 500 ppm, and calcium alginate 500 ppm are shown in Figure 2.

Infrared spectroscopy is a tool to study the state of bonds and microstructure of materials in organic chemistry—in other words, to study hydrogen bonds and other reactions in addition to the ability to combine polymers. Since different functional groups have absorption at certain frequencies and changes in the structure of materials cause changes in absorption frequencies, IR spectroscopy is introduced as a suitable tool for detecting and displaying structural changes in the method. As can be seen, the general structure of the peaks for both samples is very similar to each other. The ones observed in 623.44 and 777.81 are related to the off-plane bends of C-H bonds of phenolic rings [51]. The peaks of 817.80 and 819.56 are related to the asymmetric tensile vibrations of P-O and observed in 1165.18 are related to the asymmetric tensile vibrations of C=O. The peak in 1376.69 in calcium alginate is related to symmetric bending vibration C-H, which is not present in chitosan. The peaks of 1405.62 and 1459.05 correspond to the flexural vibrations of C-H, and the peaks of 1650.13 show the tensile vibrations of the C=C double bonds, which indicate the helical structure of the second wave. The peak of 1744.74 tensile vibrations is C=O, and the peaks observed in area 2856 belong to the CH_2_ and CH_3_ groups of symmetric tensile vibrations. The peaks in the 2924.95 and 1326.1326 CH_2_ groups are asymmetric tensile vibrations of the methylene groups in the phenolic rings, and the peak of 30.77 is related to the tensile vibrations =C-H. The chitosan spectrum shows a strong and wide peak at 3433.54, which in calcium alginate has reduced the intensity of this peak and indicates the overlap caused by the bonding of hydrogen bonds by the tensile vibrations of O-H and N-H.

### 3.5. Effect of Encapsulation on In Vitro Gas Production

The gas production of the experimental treatments at different incubation times is presented in Table 3.

At the 2 h incubation, the treatment containing chitosan (7%) had the highest gas production among the experimental and control treatments. However, this increase in gas production was not significant, and the treatments containing flaxseed oil (14%) and chitosan (14%) had significantly lower gas production compared to control and other treatments (*p* < 0.05). During incubation times 4 to 12 h, the treatment containing calcium alginate (14%) had the highest gas production compared to the control treatment and other treatments, and this increase in gas production was also statistically significant (*p* < 0.05). At the incubation times, 16 to 96 h, the treatment containing flaxseed oil (7%) had more gas production among the treatments, and after that, the treatment containing calcium alginate (14%) had more than the control treatment and other treatments (*p* < 0.05). Additionally, the lowest gas production during the incubation period (2–96 h) is for treatment containing flaxseed oil (14%).

### 3.6. The Effect of Flaxseed oil Encapsulation on the In Vitro Digestibility of Dairy Cattle Diets

The effect of flaxseed oil encapsulation on the in vitro digestibility of dairy cattle diets is shown in Table 4.

After 24 h of incubation, the results show that the experimental treatments had less dry matter digestibility than the control treatment and only the treatment containing calcium alginate (14%) significantly increased the digestibility compared to the control treatment and the treatment containing Chitosan (7%) had the lowest dry matter disappearance among experimental treatments (*p* < 0.05). After 2 h of incubation, the highest rate of organic matter disappearance was related to the control treatment, but with the incubation process continuing until 24, the treatment containing calcium alginate (14%) had the highest digestibility of organic matter. The lowest levels at incubation times of 2, 4, 8, and 12 were related to treatments containing chitosan (7%), calcium alginate (7%), flaxseed oil (14%), and chitosan (14%) (*p* < 0.05).

The crude protein disappearance of the experimental treatments is presented in Table 4, the difference between which was statistically significant with the control treatment (*p* < 0.05). During 24 h of incubation, the treatment containing flaxseed oil (14%) had the highest crude protein digestibility compared to the control and other treatments. The lowest rate of crude protein disappearance related to treatments containing chitosan (7%) (*p* < 0.05).

The results of the in vitro digestibility of neutral detergent fiber and acid detergent fiber are presented in Table 4. According to the presented results, 2 h after incubation, the highest rate of digestibility of neutral detergent fiber is related to the treatment containing chitosan (14%), and the lowest rate is related to the treatment containing flaxseed oil (14%). At 4 h after incubation, the treatment containing calcium alginate (14%) had the highest, and the treatment containing chitosan (7%) had the lowest rate of fiber digestibility. This trend changed in 8 h after incubation, and the treatment containing chitosan (7%) had the highest rate, and the treatment containing flaxseed oil (14%) after the control treatment had the lowest rate among the treatments (*p* < 0.05). At 12 and 24 h after incubation, the rate of disappearance of insoluble fibers in the neutral detergent changed again, and this time the treatment containing calcium alginate (14%) had the highest amount, and the treatments containing flaxseed oil (14%) and chitosan (7%) had the lowest rate (*p* < 0.05). The presented results on the disappearance of acid detergent fiber show that at 2 and 4 h after incubation, the treatment containing calcium alginate (14%) has the highest digestibility of acid detergent fiber detergent. However, 8 h after incubation, this trend changed, and the treatment containing calcium alginate (7%) had the highest amount, and at 12 h after incubation, the treatment containing flaxseed oil (14%) had the highest digestibility (*p* < 0.05). Finally, at 24 h after incubation, the treatment containing calcium alginate (14%) caused a significant increase in the digestibility of acid detergent fiber compared to other experimental treatments (*p* < 0.05).

### 3.7. The Effect of Flaxseed Oil Encapsulation on Biohydrogenation of Fatty Acids in Experimental Treatments

The effect of flaxseed oil encapsulation on in vitro ruminal biohydrogenation of fatty acids of experimental treatments is presented in Table 5. The results show that the percentage of saturated fatty acids in the treatment containing calcium alginate (7%) during incubation time increased significantly compared to the control treatment and other treatments, while the percentage of unsaturated fatty acids in this treatment showed a significant decrease (*p* < 0.05). Encapsulation of flaxseed oil with chitosan reduces the hydrogenation of saturated fatty acids so that the treatment containing chitosan (14%) at 2 and 4 h of incubation and the treatment containing chitosan (7%) at 24 h of incubation increases the percentage of unsaturated fatty acids and the amount of saturated fatty acids significantly reduced (*p* < 0.05). Treatments containing calcium alginate (7% and 14%) and treatments containing flaxseed oil (7% and 14%) significantly reduced the percentage of unsaturated fatty acids compared to the control treatment. Encapsulation reduced the percentage of short-chain fatty acids after 24 h of incubation for treatment with chitosan (7%), but treatment with calcium alginate (7%) increased it significantly. The percentage of medium-chain fatty acids among the experimental treatments was significantly higher than the control treatment, and its highest amount was found in 2 h of incubation with chitosan (14%) and in 4 h of incubation treatment with calcium alginate (7%), with no significant difference between chitosan (7%) and flaxseed oil (14%).

Encapsulation of flaxseed oil protects long-chain unsaturated fatty acids from ruminal biohydrogenation so that the percentage of these fatty acids in the treatment containing chitosan (7%) increased significantly (*p* < 0.05), and the treatment containing calcium alginate (7%) after 24 h of incubation reduced the percentage of these fatty acids compared to the control treatment and other treatments.

## 4. Discussion

### 4.1. Capsulation Efficiency

The micro coating efficiency was 87.47% for 500 ppm chitosan, 67.45% for 1000 ppm chitosan, 74.5% for 500 ppm calcium alginate, and 53.28% for 1000 ppm calcium alginate. The efficiency of the micro coating is to determine the amount of oil that has been successfully coated and is calculated from the amounts of surface oil and total oil. This parameter is one of the important factors in determining the stability of micro coated compounds because it indicates the presence of oil on the surface of powder particles and the ability of the walls to prevent the release of internal oil [52]. In general, there is a view that the stability of the compounds increases with increasing the efficiency of micro coating, and in order to achieve optimal conditions, one should try to increase the efficiency of micro coating as much as possible [53,54]. Previous studies have shown that wall, core types, emulsion properties, and drying parameters can affect the performance of micro coating [52,55,56,57,58]. Swetank et al. [59] observed that the combination of two wall materials (protein and polysaccharide) reduces the efficiency of the micro coating compared to the use of each alone. The main factors affecting the micro coating efficiency of micro coating oils and flavors are the type of wall material, the properties of the core material (concentration and volatility), the properties of the emulsion (total solids, viscosity, and particle size), and the drying conditions, thus optimizing the drying process. Liu et al. [60] showed that in flaxseed oil microencapsulation by compound aggregation method, increasing the homogenizer speed to 9000 increases productivity. Additionally, in another study, the efficiency of microencapsulation of drug compounds in chitosan coatings by emulsion method was very high and in the range of 52.2 to 80.1% for different treatments [61].

### 4.2. Zeta Potential

The significantly higher zeta potential of the prepared nanoparticles indicates excellent colloidal dispersion stability. The positive zeta potential also enhances the formation of non-stoichiometric nanoparticles [62]. The higher zeta potential means that the treatment has good conditions for emulsion surface charge and electrostatic repulsion between the particles, which prevents the particles from sticking together and clumping in the emulsion. Surface charge, also known as the zeta potential, affects encapsulation efficiency, colloidal stability, and particle interaction with the cell and the environment. The high zeta potential of colloidal particles increases the electrostatic repulsion force and thus increases the physical stability of the system. Various factors such as ionic strength, type and concentration of polysaccharide and protein biopolymers used, and the ratio between them on the effective surface charge and zeta-complex potential are effective [63].

### 4.3. Particle Size Distribution

The accumulation of non-stoichiometric chitosan nanoparticles is probably due to the presence of more than one chemical. Of course, there is also the possibility of self-massification [64]. Additionally, the diagram of the particle size distribution of microcapsules after formation is shown in Figure 1. Dynamic Light Scattering (DLS) is a physical method that uses particles, particles, and particles in all directions. Reducing the particle size to the nanometer scale increases the desired properties such as stability, transparency, and encapsulation efficiency of the system. The results of research by Swetank, Karthik, and Anandharamakrishnan [59] on vanilla oil micro coated with different methods show that both wall materials and micro coating techniques significantly affect the shape, size, and overall structure of microcapsules.

In the study of Soottitantawat, Bigeard, Yoshii, Furuta, Ohkawara, and Linko [51], it was reported that the particle size of the emulsion has a very significant effect on the preservation of flavorings. According to Calvo et al. [65], the particle size obtained depends on the concentration of chitosan and alginate polymer, so when the concentration of chitosan and alginate is low, the particle size will be smaller. The higher the alginate, the larger the particle size produced, and under the same conditions, as the concentration of biopolymers increases, the particle size increases. Hosseini, Zandi, Rezaei, and Farahmandghavi [39], for their research with the method of particle oil extract of oregano essential oil in azan, have been used in the treatment of acne. The data of the weighting particles and the charged particles in the chitosan range from 40 to 80 nm. The weight of nanoparticles was greater than that of oregano essential oil particles. In an experiment, micro coatings using soy isolate on the wall on the micro coating efficiency of fish oil stated that the presence of alginate in the wall increases the particle size [66].

### 4.4. Effect of Encapsulation on In Vitro Gas Production

The volume of gas produced increases as it approaches the end of the incubation time because in the gas production test method, the microbial population is still stable at all hours of incubation, and there is no entry and exit for microorganisms. However, as incubation progresses, some microorganisms die and become an additional substrate for other microorganisms. Eventually, this process leads to an increase in cumulative gas production at the end of the incubation period [67]. In a study by Sinclair et al. [68] on the effect of flaxseed oil and fish oil on gas production, it was observed that treatments containing flaxseed oil and fish oil produced less gas than the control group due to the presence of linolenic acid in this oil, which was consistent with the results of this experiment, and the treatment containing octane oil (14%) had lower gas production. Additionally, in the gas production and batch culture experiments of Safari et al. [69], statistical comparison of treatments with protected fish oil compared to treatments containing unprotected fish oil showed better performance of encapsulated treatments. Their experiments showed that the total gas production and gas production in the first 24 h of in vitro incubation was significantly reduced by adding unprotected fish oil to the diet. Da Silva et al. [70] showed that adding 3.5% sunflower oil increased in vitro gas production but did not affect methane production. Differences in the results of different experiments are due to differences in the source of fat used, percentage of fat used, ruminant species, experimental conditions, used rations, and base substrate. Several investigations on the inclusion of unsaturated oils have indicated that it has an adverse effect on fiber digestion and the rumen bacterial population [71,72]. In general, by adding less than 10% fat to the diet, the digestibility of structural carbohydrates in the rumen is reduced by about 50% or even more. This decrease in digestibility is accompanied by a decrease in the production of volatile fatty acids and methane and hydrogen gases [73].

### 4.5. The Effect of Flaxseed Oil Encapsulation on the In Vitro Digestibility of Dairy Cattle Diets

Supplementation diet with flaxseed oil at levels of 7, 14% decreased DM digestibility compared to control (*p* < 0.05). The results of Zinn et al. [74] showed that using unsaturated fat at the level of 6% of the dry matter in the diet of fattening calves reduced the rumen digestion of organic matter insoluble fibers in neutral detergent. The results of a study on fattening lambs showed that the digestibility of nutrients, including organic and crude protein, was significantly affected by the treatment containing unsaturated fatty acids [75]. The data obtained from the study of Mansuri, Nikkhah, Rezaeian, Moradi Shahrbaback, and Mirhadi [67] are consistent with the present study results. Many researchers have reported that the addition of oil negatively affects dry matter digestion [76]. Machmüller et al. [77] added coconut oil to the lambs’ diets and found that methane production was reduced by 26% without affecting digestibility. Machmüller et al. [78] also reported that when coconut oil is used in the diet of ruminants, it has no effect on the absorption of all nutrients in the gastrointestinal tract but reduces the activity of ruminal methanogenesis. It has been confirmed that the total fat in the diet should not exceed 6 to 7% of dry matter because it negatively affects digestion and absorption of nutrients [79]. According to Jordan, Kenny, Hawkins, Malone, Lovett, and O’Mara [76], adding high levels (24% dry matter) of coconut oil to the diet of fattened cows fed 50% forage and 50% concentrate reduced the digestibility and consumption of the diet, but lower levels of oil (between 10–28% dry matter) did not affect these indicators. Decreased digestibility by encapsulating treatments can be explained by factors such as the effects of fat coating, reduced cellulolytic bacteria, and inability to bind to fibers. The different effects of different microcapsule sources on different digestibility parameters can be due to differences in the amount of oil released from different microcapsules per unit time and the total amount of oil released from the microcapsules. A study reported that rumen-protected fat supplementation did not affect dry matter, organic matter, crude protein, neutral, or acidic insoluble fibers in sheep [80]. In contrast, Bhatt and Sahoo [81] reported higher organic matter digestibility with rumen-protected fat supplementation. The inclusion of chitosan in the diet reduced nutrient digestibility. This reduction in nutrient digestibility was likely due to antimicrobial action of chitosan against ruminal microbes (protozoa and fibrolytic bacteria) [82]. Protozoa play an important role in protein degradation in ruminant [83].

### 4.6. The Effect of Flaxseed Oil Encapsulation on Biohydrogenation of Fatty Acids in Experimental Treatments

Fatty acid protection methods, using the method of making calcium salts and coated calcium salts, eliminated the negative effect of unsaturated fatty acids on the parameters of the cell wall, dry matter, and organic matter digestibility. They did not have a negative effect on fat digestibility and fat supplements. This indicated that the type of coating used was appropriate. Chouinard et al. [84] investigated the effect of calcium salts of three types of canola, soybean, and flaxseed oils on nutrient digestibility. They found that none of them had a significant effect on NDF and ADF digestibility and increased dry matter, organic matter, and protein digestibility.

Jenkins and Fotouhi [85] reported that the addition of flaxseed oil reduced protein degradability compared to the control group (*p* < 0.05). The different effects of different types of unsaturated fatty acids on the degradability parameters can be considered as the effect of their unsaturation and the subsequent effects on the microbial population of the gastrointestinal tract. In addition, the different rate and extent of biogenic hydrogenation of ruminal fatty acids may explain some of this effect [86]. By inhibiting bacterial proteases, the active and effective compounds in essential oils reduce protein digestion in the rumen and their use in the intestine. After absorption in the small intestine, it is effectively used in the body of ruminants and improves animal production efficiency. Schauff and Clark [87] stated that supplementation of calcium salt of long-chain fatty acid in dairy animals increased the crude protein digestibility.

Geraeily [88] reported that flaxseed oil increased the digestibility of insoluble fibers in neutral detergents. Schroeder et al. [89] reported that adding your grain with different treatments did not significantly affect the digestibility of NDF and ADF. In studies on unsaturated fats, this type of reduction in digestibility has been reported as a result of the negative effect of unsaturated fatty acids on cellulose-degrading microorganisms and the physical coating of feed particles, and the prevention of microorganisms from adhering to that [90]. However, unsaturated fats inhibit gram-positive bacteria that break down cellulose [91].

Encapsulation of flaxseed oil protects long-chain unsaturated fatty acids from ruminal biohydrogenation so that the percentage of these fatty acids in the treatment containing chitosan (7%) increased significantly (*p* < 0.05) and the treatment containing calcium alginate (7%) after 24 h of incubation, reducing the percentage of these fatty acids compared to the control treatment and other treatments. The antimicrobial properties of chitosan may have prevented the growth of microorganisms and, consequently, prevented ruminal biohydrogenation. Yuan et al. [92]’s research on the antimicrobial and antioxidant activity of chitosan coatings and films containing essential oils and their effectiveness in feeding systems. Their research showed that the combination of essential oils significantly increased the antimicrobial, antioxidant, and fungal properties of chitosan films. These films are commonly used to increase shelf life and reduce lipid oxidation in fish and meat products. Glasser et al. [93] stated that increasing the concentration of C18:3 isomers with oilseed supplements in the form of seeds and oil is only possible to a limited extent unless protected fats are used. The linolenic fatty acid is converted to a saturated fatty acid by biohydrogenation in ruminants. The longer passage rate of forage through the rumen and the more biohydrogenation of linolenic acid compared to linoleic acid limits the amount of accessibility of this fatty acid to be absorbed into tissues [94]. Increasing the incubation time increases the apparent biohydrogenation of unsaturated fatty acids. In the study of Dohme et al. [95], by increasing the incubation time from 12 to 24 and 48 h, the amount of biohydrogenation of fatty acids in fish oil composition increased, which can be increased by increasing lipolysis and availability of non-esterified fatty acids. Increasing the incubation time from 12 to 24 and 48 h increased the rate of bio-hydrogenation in calcium salts, which can be justified by increasing the release of fatty acids from the salt structure by reducing the acidity of ruminal fluid as a result of fermentation in the culture medium.

Today, to change the fatty acid profile of cow’s milk, extensive research has been done to reduce short-chain fatty acids and increase long-chain unsaturated fatty acids. The easiest way to change the fat content of milk is to change the diet of livestock and use compounds and nutrients containing unsaturated fatty acids [13,96]. Liu et al. [97] reported that short-chain milk fatty acids (6- to 14 C fatty acids) are formed mainly by the intrathecal synthesis in mammary epithelial cells of acetate and butyrate produced in the rumen, and C:16 milk fatty acid from two ways of intra-tissue synthesis and receiving from blood. The researchers reported that increasing the amount of long-chain unsaturated fatty acids reduces the intra-tissue synthesis of short-chain fatty acids in the breast. The amount of biohydrogenation can be explained by the efficiency of micro coating, which increases the amount of surface oil and decreases the efficiency of micro coating, increases the access of microbial enzymes to the oil in the microcapsules, and ultimately increases lipolysis and biohydrogenation. In general, unlike calcium salt technology, which involves chemical changes in the structure of a fatty acid to make the carboxyl group inaccessible, in microencapsulation, the goal is to use physical protection by creating a suitable coating around the fatty acid source in order to reduce the availability of microorganisms or microbial and plant enzymes in the rumen environment with fatty acids and ultimately increase the passage of unsaturated fatty acids into the small intestine.

Khalili et al. [98] investigated the encapsulation of thyme oil in a chitosan-benzoic acid nanogel that may have increased antimicrobial activity against *Aspergillus flavus*. Observation of the obtained results showed that chitosan-benzoic acid nanogel has a synergistic effect on the antimicrobial properties of thyme. In addition, because essential oils are volatile and unstable, encapsulation by this nanogel significantly increased its shelf life and antimicrobial properties. Szumacher-Strabel et al. [99] studied the effect of linoleic acid-rich oils on rumen fermentation parameters in sheep, goats, and dairy cows using the Batch culture system. They also showed that saturated fatty acids did not decrease numerically. While the amount of unsaturated fatty acids in dairy cows’ ruminal fluid increased numerically, it was not significant in the ruminal fluid of sheep and goats after fermentation. In experiments performed by Elnashar et al. [100] on the encapsulation method to protect unsaturated fatty acids from in vitro ruminal biohydrogenation, the encapsulation process had no significant effect on the polyunsaturated fatty acids (PUFA) fraction. Their results showed no significant difference between the fatty acid content of flaxseed oil before and after the encapsulation process. After encapsulation in the batch culture system, the total content of unsaturated and rumen saturated fatty acids in flaxseed decreased. In a recent laboratory study, a high protective effect (99%) of rumen microbes was reported for flaxseed oil encapsulated with hydrogenated palm oil after 8 h of incubation [101]. According to Khalilvandi-Behroozyar et al. [102], coating of calcium salts in fish oil using saturated fatty acids increased the amount of total fatty acids and increased the ratio of saturated fatty acids to unsaturated fatty acids. The use of calcium salts in fish fatty acids significantly reduced the bio-hydrogenation of unsaturated fatty acids compared to unprotected sources. Comparison of the rate of bio-hydrogenation shows the appropriate efficiency of calcium salts in reducing the rate of bio-hydrogenation of unsaturated fatty acids in fish oil. However, coating increased the protective effect of calcium salts. In addition, there was a significant difference between the rate of bio-hydrogenation of coated supplements with different amounts of coating material. Certainly, this study was conducted in vitro, and the results obtained are to be considered valid as described. Future research will also have to be able to deepen these aspects in vivo, performing studies in different ruminant species.

## 5. Conclusions

It was concluded that chitosan and calcium alginate at 500 ppm is suitable for encapsulating flaxseed oil with high encapsulation efficiency. Encapsulation of flaxseed oil with calcium alginate (14%) increased gas production (compared to control and flaxseed oil (14%)), the disappearance of dry matter, organic matter, and crude protein, NDF and ADF. Encapsulation of flaxseed oil with chitosan (7%) reduced hydrogenation of rumen unsaturated fatty acids. Encapsulation of flaxseed oil with calcium alginate (7% and 14%) increased saturated fatty acids.

## Figures and Tables

**Table 1:** Ingredients and chemical composition of the experimental diet (based on the dry matter).

Diet Ingredients	Content (%)
Alfalfa hay	21
Corn silage	19
Beet pulp	7
Wheat bran	2
Concentrate	51
*Chemical Composition*	
Dry matter	59
Organic matter	92.86
Crude protein	20.3
Neutral detergent fiber	38.57
Acid detergent fiber	20.00

**Table 2:** Microcoating efficiency, zeta potential, and particle size of chitosan 500 ppm and 1000 ppm and calcium alginate 500 ppm and 1000 ppm.

	Efficiency (%)	Zeta Potential (mv)	Particle Size (nm)
chitosan 500 ppm	87.47	+56.2	190.2
chitosan 1000 ppm	67.45	+45.5	-
calcium alginate 500 ppm	74.50	+0.9	334
calcium alginate 1000 ppm	53.28	+0.6	-

**Table 3:** The effect of flaxseed oil encapsulation on total gas production of dairy cattle diet in incubation times (mL/g dry matter).

Treatments	Incubation Times (h)										
	2	4	6	8	12	16	24	36	48	72	96
Control	5.52 ^a^	15.04 ^b,c^	26.79 ^b,c^	38.29 ^a,b,c^	56.77 ^b^	74.45 ^c,d^	93.69 ^b,c^	109.42 ^d^	120.31 ^c^	126.30 ^c^	130.41 ^c^
flaxseed oil (7%)	5.39 ^a^	17.70 ^a,b^	30.19 ^a,b^	42.08 ^a,b^	69.56 ^a^	98.83 ^a^	116.18 ^a^	138.90 ^a^	147.93 ^a^	153.66 ^a^	156.44 ^a^
flaxseed oil (14%)	2.79 ^b^	11.89 ^c^	20.33 ^c^	31.29 ^c^	46.12 ^c^	63.80 ^d^	75.84 ^d^	88.31 ^e^	94.81 ^d^	98.34 ^d^	99.72 ^d^
Chitosan (7%)	6.19 ^a^	15.11 ^b,c^	26.13 ^b,c^	37.22 ^b,c^	54.24 ^b,c^	70.92 ^c,d^	88.61 ^c,d^	115.83 ^c,d^	127.55 ^b,c^	132.62 ^bc^	135.79 ^b,c^
Chitosan (14%)	3.66 ^a,b^	17.64 ^a,b^	28.59 ^a,b^	40.09 ^a,b,c^	64.72 ^a,b^	87.82 ^a,b^	103.93 ^a,b^	129.98 ^a,b,c^	138.28 ^a,b^	146.93 ^a,b^	150.78 ^a,b^
Calcium alginate (7%)	5.52 ^a^	16.79 ^b,c^	28.94 ^a,b^	42.90 ^a,b^	61.19 ^a,b^	81.53 ^b,c^	98.17 ^b,c^	122.22 ^b,c,d^	131.85 ^a,b,c^	139.84 ^a,b,c^	144.02 ^a,b,c^
Calcium alginate (14%)	5.46 ^a^	22.38 ^a^	35.27 ^a^	47.29 ^a^	70.38 ^a^	92.79 ^a,b^	107.22 ^a,b^	133.08 ^a,b^	140.24 ^a,b^	148.50 ^a,b^	153.21 ^a,b^
SEM	0.788	1.711	2.391	2.927	3.371	4.204	4.426	5.098	5.420	5.924	6.040
*p*-value	0.053	0.0096	0.0094	0.022	0.0002	<0.0001	<0.0001	<0.0001	<0.0001	<0.0001	<0.0001

Treatments: Control: diet without supplemented oil, flaxseed oil (7%): diet containing 7% of flaxseed oil, flaxseed oil (14%): diet containing 14% of flaxseed oil, Chitosan (7%): diet containing 7% flaxseed oil encapsulated with 500 ppm chitosan, Chitosan (14%): diet containing 14% flaxseed oil encapsulated with 1000 ppm chitosan, Calcium alginate (7%): diet containing 7% flaxseed oil encapsulated with 500 ppm calcium alginate, Calcium alginate (14%): diet containing 14% flaxseed oil encapsulated with 1000 ppm calcium alginate. SEM: Standard error of means. ^a,b,c,d,e^ Means within the same column with different superscripts differ significantly (*p* < 0.05).

**Table 4:** The effect of flaxseed oil encapsulation on average in vitro digestibility of dairy cattle diet in incubation times (% of DM).

Treatments	Incubation Times (h)				
	2	4	8	12	24
*Dry matter digestibility*					
Control	32.42 ^a^	34.85 ^b^	49.81 ^a^	51.18 ^b^	61.10 ^b^
flaxseed oil (7%)	25.99 ^b^	32.44 ^b^	37.54 ^d^	45.76 ^c^	49.23 ^d^
flaxseed oil (14%)	32.40 ^a^	33.87 ^b^	39.54 ^cd^	44.21 ^c,d^	53.73 ^c^
Chitosan (7%)	21.98 ^c^	23.74 ^c^	36.60 ^d^	42.56 ^d^	46.35 ^d,e^
Chitosan (14%)	25.61 ^b^	33.73 ^b^	37.25 ^d^	42.07 ^d^	43.58 ^e^
Calcium alginate (7%)	23.11 ^b,c^	24.66 ^c^	42.60 ^b,c^	45.76 ^c^	74.40 ^a^
Calcium alginate (14%)	33.55 ^a^	38.93 ^a^	45.75 ^b^	54.32 ^a^	75.06 ^a^
SEM	1.007	0.966	1.162	0.729	1.233
*p*-value	<0.0001	<0.0001	<0.0001	<0.0001	<0.0001
*Organic matter digestibility*					
Control	39.71 ^a^	44.36 ^b^	50.50 ^b,c^	55.49 ^a,b,c^	61.84 ^a,b,c^
flaxseed oil (7%)	31.19 ^c^	42.50 ^b^	47.46 ^c,d^	54.28 ^b,c^	58.03 ^c,d^
flaxseed oil (14%)	28.01 ^d^	43.07 ^b^	45.88 ^d^	58.10 ^a,b^	60.33 ^b,c^
Chitosan (7%)	20.95 ^e^	29.22 ^c^	47.65 ^b,c,d^	57.83 ^a,b^	61.80 ^a,b,c^
Chitosan (14%)	36.14 ^b^	49.87 ^a^	51.13 ^b^	52.42 ^c^	54.71 ^d^
Calcium alginate (7%)	22.22 ^e^	26.20 ^c^	47.01 ^c,d^	58.57 ^a,b^	63.57 ^a,b^
Calcium alginate (14%)	31.59 ^c^	51.18 ^a^	55.61 ^a^	60.30 ^a^	66.62 ^a^
SEM	0.743	1.678	1.107	1.622	1.610
*p*-value	<0.0001	<0.0001	0.0003	0.048	0.0035
*Crude protein digestibility*					
Control	27.72 ^c^	48.88 ^c^	53.99 ^b^	59.30 ^b^	67.71 ^a^
flaxseed oil (7%)	40.59 ^b^	45.39 ^c,d^	54.47 ^b^	67.55 ^a^	68.58 ^a^
flaxseed oil (14%)	37.20 ^b^	53.70 ^b^	63.40 ^a^	66.81 ^a^	69.85 ^a^
Chitosan (7%)	19.37 ^d^	43.01 ^d^	49.29 ^c^	50.73 ^d^	63.60 ^b^
Chitosan (14%)	38.86 ^b^	52.71 ^b^	55.42 ^b^	61.06 ^b^	69.70 ^a^
Calcium alginate (7%)	31.45 ^c^	36.77 ^e^	49.29 ^c^	55.08 ^c^	57.36 ^c^
Calcium alginate (14%)	50.97 ^a^	63.35 ^a^	66.38 ^a^	67.79 ^a^	69.59 ^a^
SEM	1.623	1.148	1.048	0.819	0.681
*p*-value	<0.0001	<0.0001	<0.0001	<0.0001	<0.0001
*NDF digestibility*					
Control	37.30 ^b^	46.78 ^a,b^	47.42 ^c^	54.88 ^b^	64.57 ^a^
flaxseed oil (7%)	28.25 ^c^	43.51 ^a,b,c^	50.35 ^a,b^	54.74 ^b^	57.67 ^b^
flaxseed oil (14%)	27.09 ^c^	46.54 ^a,b^	47.14 ^b,c^	53.10 ^b^	55.52 ^b,c^
Chitosan (7%)	38.16 ^b^	38.51 ^c^	52.39 ^a^	53.64 ^b^	54.71 ^c^
Chitosan (14%)	45.95 ^a^	46.95 ^a,b^	52.12 ^a,b^	54.20 ^b^	55.08 ^c^
Calcium alginate (7%)	38.98 ^b^	42.32 ^b,c^	49.51 ^a,b^	57.11 ^a,b^	66.85 ^a^
Calcium alginate (14%)	35.76 ^b^	47.85 ^a^	51.85 ^a,b^	62.81 ^a^	65.97 ^a^
SEM	1.452	1.616	2.430	1.885	0.799
*p*-value	<0.0001	0.013	0.035	0.035	<0.0001
*ADF digestibility*					
Control	23.96 ^c^	31.65 ^b^	42.83 ^a,b,c^	52.83 ^a,b^	57.30 ^b^
flaxseed oil (7%)	28.20 ^b^	33.05 ^b^	39.94 ^b,c^	52.64 ^a,b^	57.11 ^b^
flaxseed oil (14%)	10.49 ^d^	34.87 ^b^	45.06 ^a,b^	56.27 ^a^	58.05 ^b^
Chitosan (7%)	23.34 ^c^	25.33 ^c^	36.94 ^c^	45.85 ^c^	51.24 ^c^
Chitosan (14%)	31.22 ^b^	33.94 ^b^	36.69 ^c^	46.08 ^c^	51.39 ^c^
Calcium alginate (7%)	30.41 ^b^	33.46 ^b^	48.31 ^a^	52.62 ^a,b^	55.60 ^b^
Calcium alginate (14%)	37.36 ^a^	39.58 ^a^	44.60 ^a,b,c^	49.43 ^b,c^	63.20 ^a^
SEM	1.032	1.215	2.504	1.824	1.193
*p*-value	<0.0001	<0.0001	0.035	0.0098	<0.0001

Treatments: Control: diet without supplemented oil, flaxseed oil (7%): diet containing 7% of flaxseed oil, flaxseed oil (14%): diet containing 14% of flaxseed oil, Chitosan (7%): diet containing 7% flaxseed oil encapsulated with 500 ppm chitosan, Chitosan (14%): diet containing 14% flaxseed oil encapsulated with 1000 ppm chitosan, Calcium alginate (7%): diet containing 7% flaxseed oil encapsulated with 500 ppm calcium alginate, Calcium alginate (14%): diet containing 14% flaxseed oil encapsulated with 1000 ppm calcium alginate. SEM: Standard error of means. ^a,b,c,d,e^ Means within the same column with different superscripts differ significantly (*p* < 0.05).

**Table 5:** Effect of flaxseed oil encapsulation on in vitro biohydrogenation of fatty acids (mg/g of fat).

		Treatments								
Items	Time (h)	Control	Flaxseed Oil (7%)	Flaxseed Oil (14%)	Chitosan (7%)	Chitosan (14%)	Calcium Alginate (7%)	Calcium Alginate (14%)	SEM	*p*-Value
SFA	2	79.52 ^c^	80.31 ^c^	61.14 ^d^	56.25 ^e^	52.37 ^f^	98.60 ^a^	91.40 ^b^	0.406	<0.0001
	4	82.62 ^d^	82.86 ^d^	96.17 ^c^	59.31 ^e^	53.82 ^f^	99.11 ^a^	92.15 ^b^	0.692	<0.0001
	24	84.16 ^d^	92.98 ^a^	88.46 ^b^	61.05 ^d^	68.69 ^c^	96.00 ^a^	92.79 ^a^	0.958	<0.0001
USFA	2	20.45 ^c^	18.46 ^c^	38.63 ^b^	39.56 ^b^	42.42 ^a^	1.22 ^e^	4.92 ^d^	0.380	<0.0001
	4	17.03 ^c^	16.42 ^c^	12.19 ^d^	35.29 ^b^	40.22 ^a^	0.48 ^f^	3.91 ^e^	0.234	<0.0001
	24	15.08 ^b^	4.61 ^c^	10.74 ^b,c^	37.88 ^a^	13.93 ^b^	1.98 ^c^	3.12 ^c^	2.534	<0.0001
SCFA	2	31.85 ^d^	36.07 ^c^	15.14 ^d^	3.26 ^f^	5.39 ^e^	77.11 ^a^	40.54 ^b^	0.224	<0.0001
	4	29.75 ^e^	45.45 ^b^	39.00 ^c^	8.87 ^e^	33.95 ^d^	64.55 ^a^	34.56 ^d^	0.954	<0.0001
	24	39.66 ^d^	47.65 ^c^	30.65 ^e^	13.30 ^f^	53.82 ^b^	70.77 ^a^	36.45 ^d^	0.741	<0.0001
MCFA	2	7.42 ^f^	17.55 ^c^	16.91 ^c^	28.56 ^b^	45.61 ^a^	14.06 ^d^	12.32 ^e^	0.308	<0.0001
	4	6.60 ^e^	9.70 ^d^	25.80 ^a^	25.53 ^a^	22.87 ^b^	25.83 ^a^	16.49 ^c^	0.491	<0.0001
	24	5.92 ^f^	12.83 ^e^	19.65 ^b^	26.99 ^a^	17.22 ^c^	12.53 ^e^	14.54 ^d^	0.368	<0.0001
LCFA	2	35.45 ^d^	16.86 ^d^	48.02 ^b^	53.92 ^a^	46.56 ^b^	8.67 ^e^	41.47 ^c^	0.791	<0.0001
	4	38.24 ^c^	22.64 ^d^	23.48 ^d^	50.34 ^a^	34.91 ^c^	7.82 ^e^	45.32 ^b^	0.448	<0.0001
	24	30.79 ^c^	17.83 ^e^	30.93 ^c^	47.85 ^a^	28.88 ^d^	15.45 ^f^	40.36 ^b^	0.336	<0.0001

Treatments: Control: diet without supplemented oil, flaxseed oil (7%): diet containing 7% of flaxseed oil, flaxseed oil (14%): diet containing 14% of flaxseed oil, Chitosan (7%): diet containing 7% flaxseed oil encapsulated with 500 ppm chitosan, Chitosan (14%): diet containing 14% flaxseed oil encapsulated with 1000 ppm chitosan, Calcium alginate (7%): diet containing 7% flaxseed oil encapsulated with 500 ppm calcium alginate, Calcium alginate (14%): diet containing 14% flaxseed oil encapsulated with 1000 ppm calcium alginate. SFA: Saturated fatty acids, USFA: Unsaturated fatty acids, SCFA: Short-chain fatty acids (C4:0–C12:0), MCFA: Medium-chain fatty acids (C13:0–C17:0), LCFA: Long-chain fatty acids (C18:0–C22:0). SEM: Standard error of means. ^a,b,c,d,e,f^ Means within the same row with different superscripts differ significantly (*p* < 0.05).

## Data Availability

Not applicable.
